# Supplementary material for: Differing structures of galactoglucomannan in eudicots and non-eudicot angiosperms
Source: PLoS One. 2023 Dec 21;18(12):e0289581. doi: 10.1371/journal.pone.0289581 (PMC10735049; doi:10.1371/journal.pone.0289581)
Supplement: S1 File — (PPTX) [file pone.0289581.s001.pptx]

## Slide 1
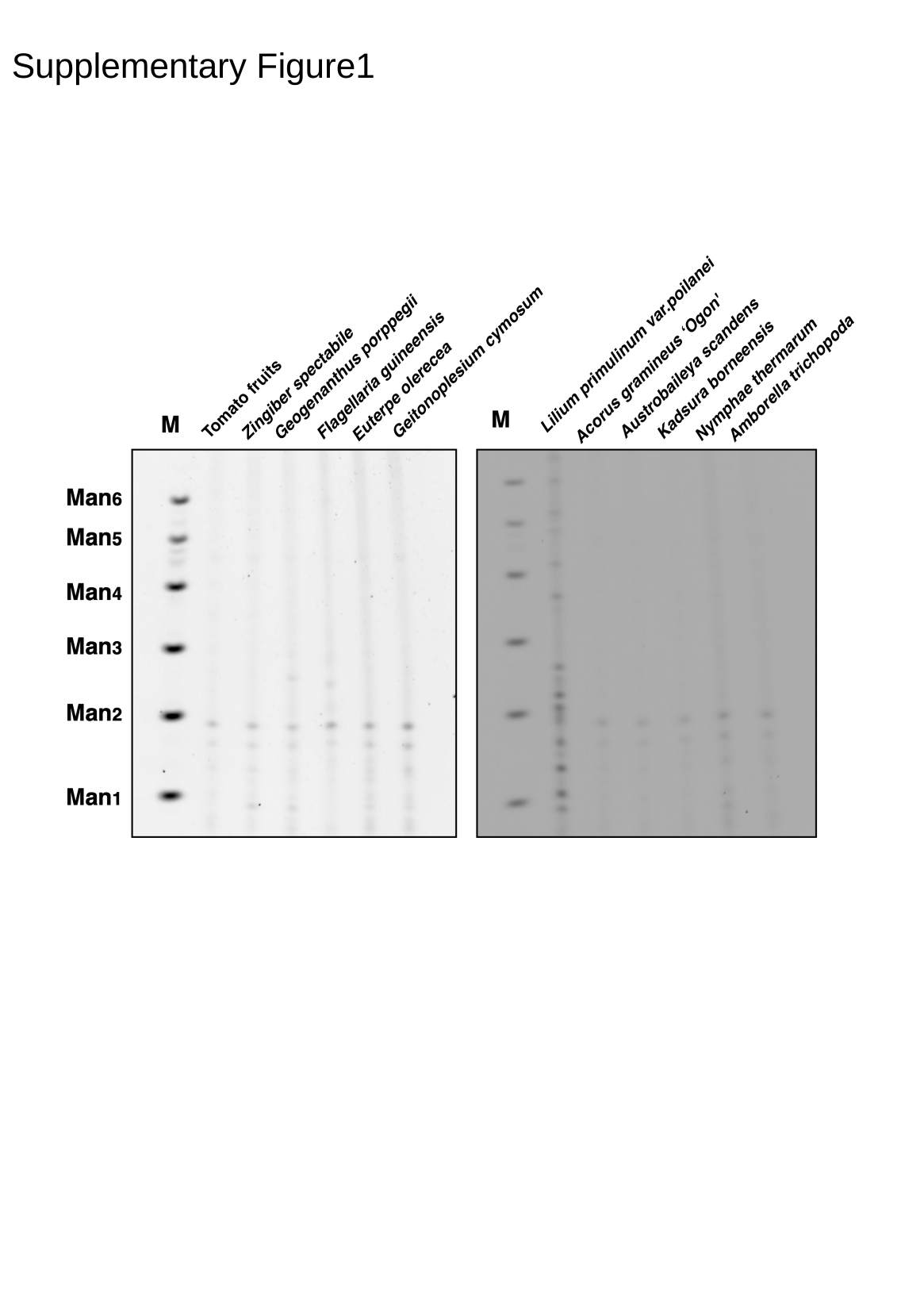

Supplementary Figure1

## Slide 2
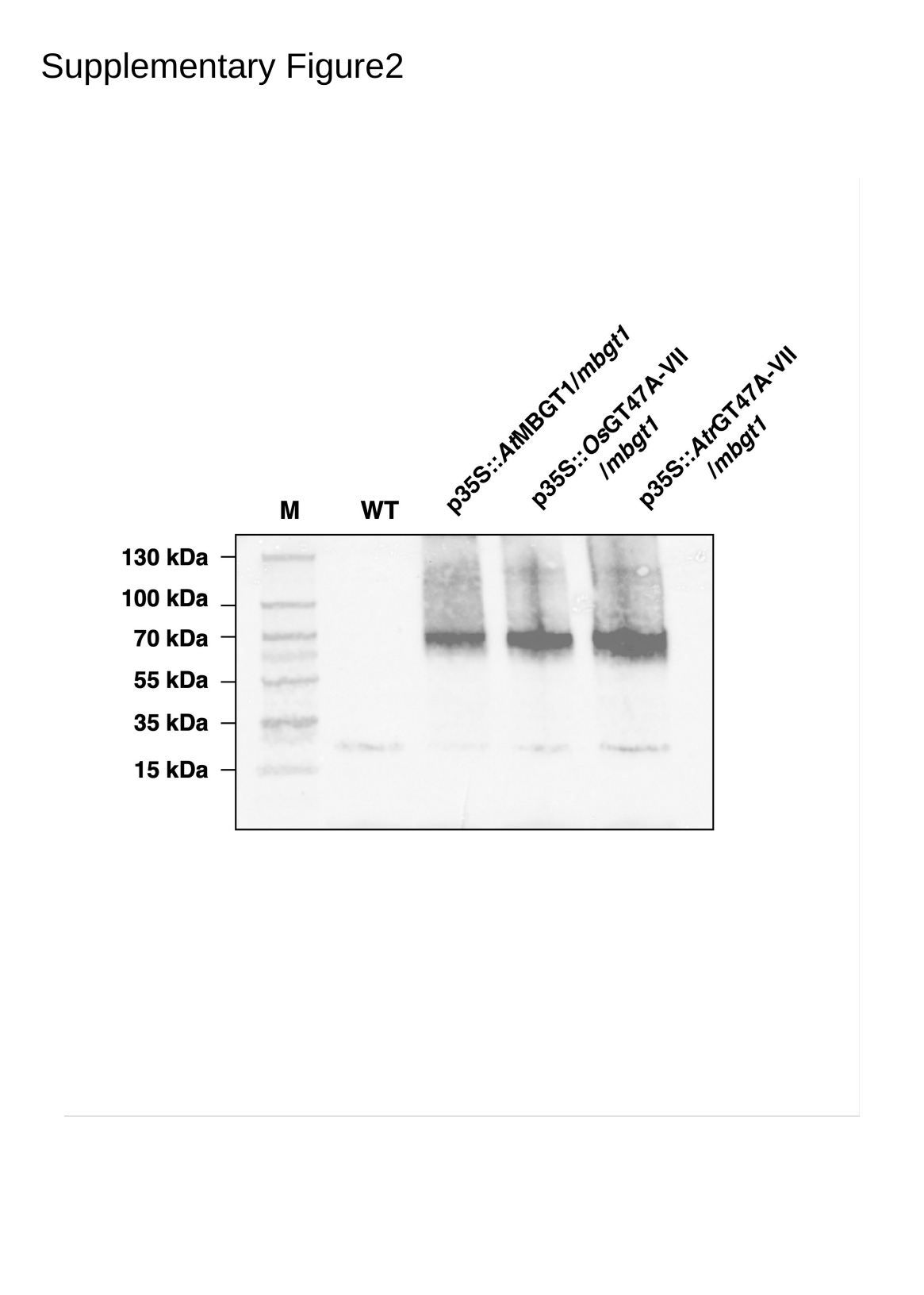

Supplementary Figure2

## Slide 3
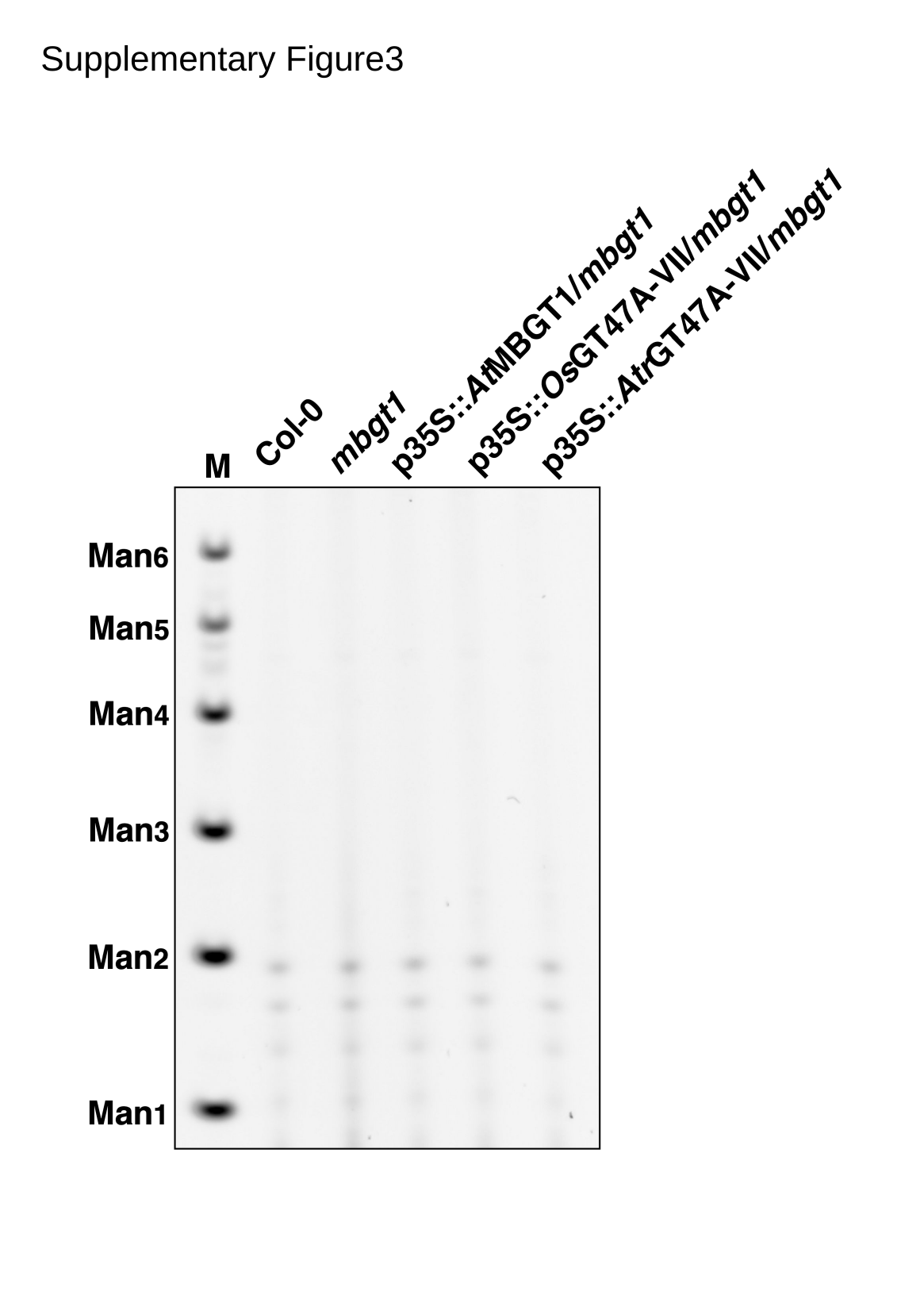

Supplementary Figure3

## Slide 4
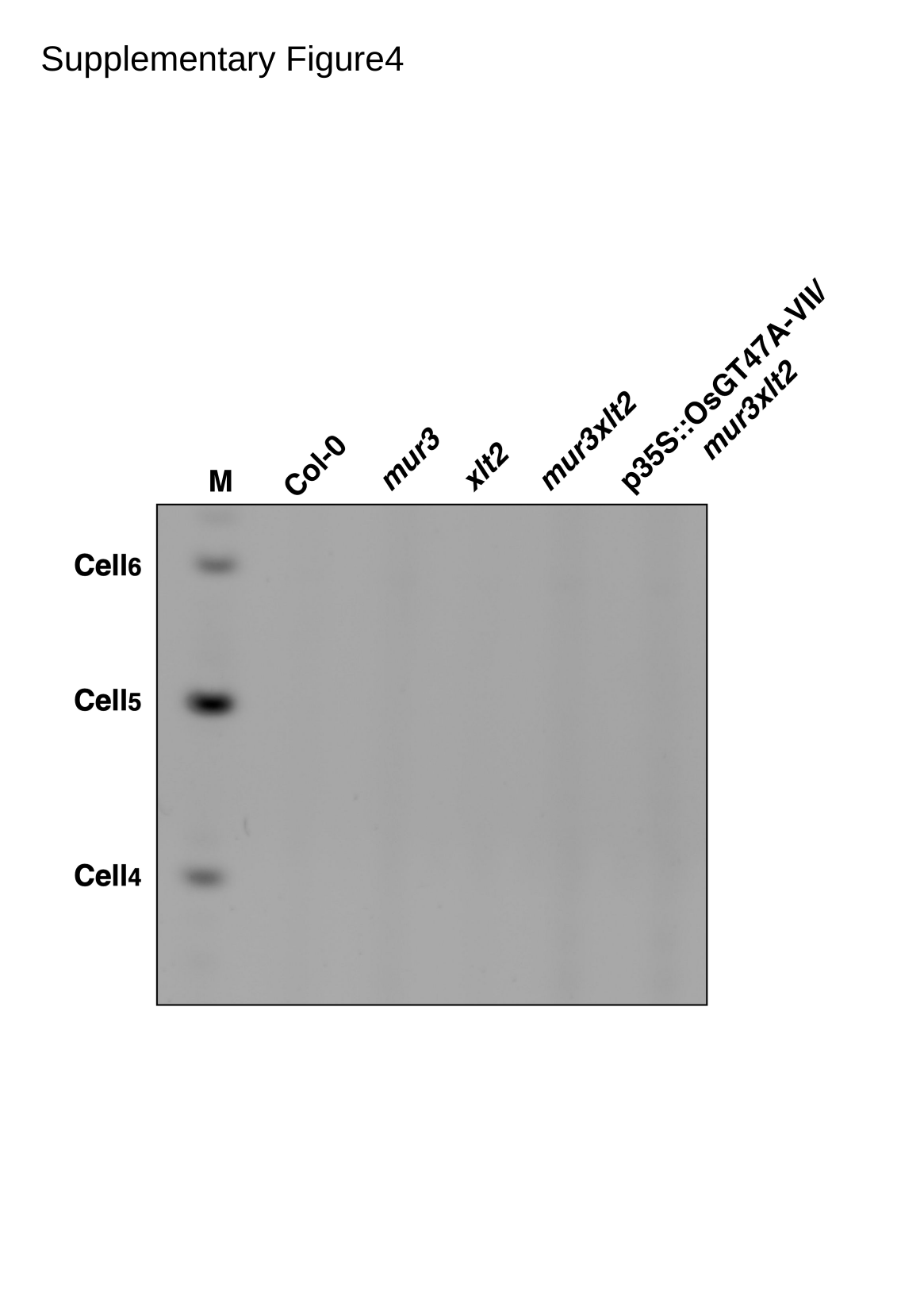

Supplementary Figure4

## Slide 5
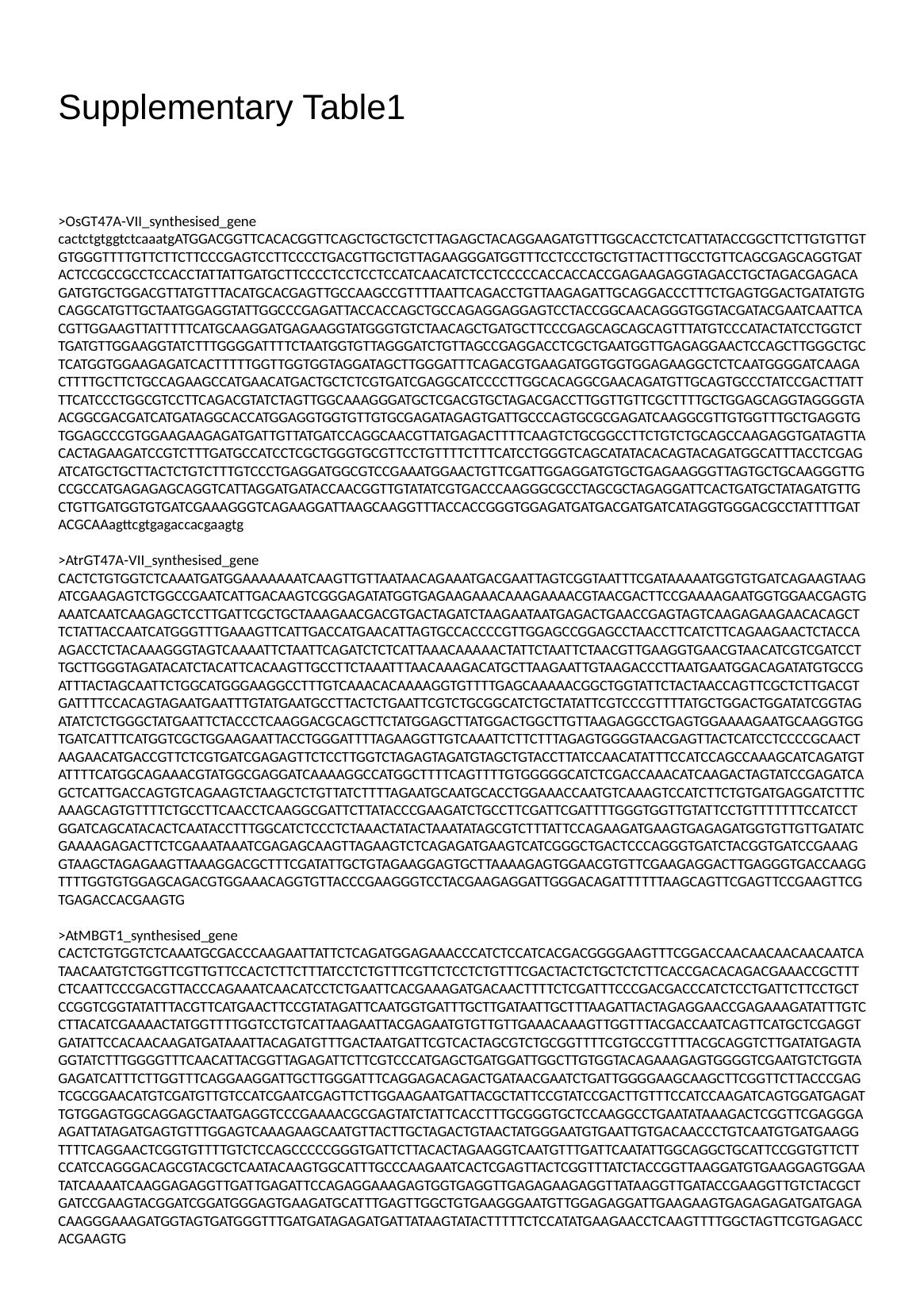

Supplementary Table1
>OsGT47A-VII_synthesised_gene
cactctgtggtctcaaatgATGGACGGTTCACACGGTTCAGCTGCTGCTCTTAGAGCTACAGGAAGATGTTTGGCACCTCTCATTATACCGGCTTCTTGTGTTGTGTGGGTTTTGTTCTTCTTCCCGAGTCCTTCCCCTGACGTTGCTGTTAGAAGGGATGGTTTCCTCCCTGCTGTTACTTTGCCTGTTCAGCGAGCAGGTGATACTCCGCCGCCTCCACCTATTATTGATGCTTCCCCTCCTCCTCCATCAACATCTCCTCCCCCACCACCACCGAGAAGAGGTAGACCTGCTAGACGAGACAGATGTGCTGGACGTTATGTTTACATGCACGAGTTGCCAAGCCGTTTTAATTCAGACCTGTTAAGAGATTGCAGGACCCTTTCTGAGTGGACTGATATGTGCAGGCATGTTGCTAATGGAGGTATTGGCCCGAGATTACCACCAGCTGCCAGAGGAGGAGTCCTACCGGCAACAGGGTGGTACGATACGAATCAATTCACGTTGGAAGTTATTTTTCATGCAAGGATGAGAAGGTATGGGTGTCTAACAGCTGATGCTTCCCGAGCAGCAGCAGTTTATGTCCCATACTATCCTGGTCTTGATGTTGGAAGGTATCTTTGGGGATTTTCTAATGGTGTTAGGGATCTGTTAGCCGAGGACCTCGCTGAATGGTTGAGAGGAACTCCAGCTTGGGCTGCTCATGGTGGAAGAGATCACTTTTTGGTTGGTGGTAGGATAGCTTGGGATTTCAGACGTGAAGATGGTGGTGGAGAAGGCTCTCAATGGGGATCAAGACTTTTGCTTCTGCCAGAAGCCATGAACATGACTGCTCTCGTGATCGAGGCATCCCCTTGGCACAGGCGAACAGATGTTGCAGTGCCCTATCCGACTTATTTTCATCCCTGGCGTCCTTCAGACGTATCTAGTTGGCAAAGGGATGCTCGACGTGCTAGACGACCTTGGTTGTTCGCTTTTGCTGGAGCAGGTAGGGGTAACGGCGACGATCATGATAGGCACCATGGAGGTGGTGTTGTGCGAGATAGAGTGATTGCCCAGTGCGCGAGATCAAGGCGTTGTGGTTTGCTGAGGTGTGGAGCCCGTGGAAGAAGAGATGATTGTTATGATCCAGGCAACGTTATGAGACTTTTCAAGTCTGCGGCCTTCTGTCTGCAGCCAAGAGGTGATAGTTACACTAGAAGATCCGTCTTTGATGCCATCCTCGCTGGGTGCGTTCCTGTTTTCTTTCATCCTGGGTCAGCATATACACAGTACAGATGGCATTTACCTCGAGATCATGCTGCTTACTCTGTCTTTGTCCCTGAGGATGGCGTCCGAAATGGAACTGTTCGATTGGAGGATGTGCTGAGAAGGGTTAGTGCTGCAAGGGTTGCCGCCATGAGAGAGCAGGTCATTAGGATGATACCAACGGTTGTATATCGTGACCCAAGGGCGCCTAGCGCTAGAGGATTCACTGATGCTATAGATGTTGCTGTTGATGGTGTGATCGAAAGGGTCAGAAGGATTAAGCAAGGTTTACCACCGGGTGGAGATGATGACGATGATCATAGGTGGGACGCCTATTTTGATACGCAAagttcgtgagaccacgaagtg
>AtrGT47A-VII_synthesised_gene
CACTCTGTGGTCTCAAATGATGGAAAAAAATCAAGTTGTTAATAACAGAAATGACGAATTAGTCGGTAATTTCGATAAAAATGGTGTGATCAGAAGTAAGATCGAAGAGTCTGGCCGAATCATTGACAAGTCGGGAGATATGGTGAGAAGAAACAAAGAAAACGTAACGACTTCCGAAAAGAATGGTGGAACGAGTGAAATCAATCAAGAGCTCCTTGATTCGCTGCTAAAGAACGACGTGACTAGATCTAAGAATAATGAGACTGAACCGAGTAGTCAAGAGAAGAACACAGCTTCTATTACCAATCATGGGTTTGAAAGTTCATTGACCATGAACATTAGTGCCACCCCGTTGGAGCCGGAGCCTAACCTTCATCTTCAGAAGAACTCTACCAAGACCTCTACAAAGGGTAGTCAAAATTCTAATTCAGATCTCTCATTAAACAAAAACTATTCTAATTCTAACGTTGAAGGTGAACGTAACATCGTCGATCCTTGCTTGGGTAGATACATCTACATTCACAAGTTGCCTTCTAAATTTAACAAAGACATGCTTAAGAATTGTAAGACCCTTAATGAATGGACAGATATGTGCCGATTTACTAGCAATTCTGGCATGGGAAGGCCTTTGTCAAACACAAAAGGTGTTTTGAGCAAAAACGGCTGGTATTCTACTAACCAGTTCGCTCTTGACGTGATTTTCCACAGTAGAATGAATTTGTATGAATGCCTTACTCTGAATTCGTCTGCGGCATCTGCTATATTCGTCCCGTTTTATGCTGGACTGGATATCGGTAGATATCTCTGGGCTATGAATTCTACCCTCAAGGACGCAGCTTCTATGGAGCTTATGGACTGGCTTGTTAAGAGGCCTGAGTGGAAAAGAATGCAAGGTGGTGATCATTTCATGGTCGCTGGAAGAATTACCTGGGATTTTAGAAGGTTGTCAAATTCTTCTTTAGAGTGGGGTAACGAGTTACTCATCCTCCCCGCAACTAAGAACATGACCGTTCTCGTGATCGAGAGTTCTCCTTGGTCTAGAGTAGATGTAGCTGTACCTTATCCAACATATTTCCATCCAGCCAAAGCATCAGATGTATTTTCATGGCAGAAACGTATGGCGAGGATCAAAAGGCCATGGCTTTTCAGTTTTGTGGGGGCATCTCGACCAAACATCAAGACTAGTATCCGAGATCAGCTCATTGACCAGTGTCAGAAGTCTAAGCTCTGTTATCTTTTAGAATGCAATGCACCTGGAAACCAATGTCAAAGTCCATCTTCTGTGATGAGGATCTTTCAAAGCAGTGTTTTCTGCCTTCAACCTCAAGGCGATTCTTATACCCGAAGATCTGCCTTCGATTCGATTTTGGGTGGTTGTATTCCTGTTTTTTTCCATCCTGGATCAGCATACACTCAATACCTTTGGCATCTCCCTCTAAACTATACTAAATATAGCGTCTTTATTCCAGAAGATGAAGTGAGAGATGGTGTTGTTGATATCGAAAAGAGACTTCTCGAAATAAATCGAGAGCAAGTTAGAAGTCTCAGAGATGAAGTCATCGGGCTGACTCCCAGGGTGATCTACGGTGATCCGAAAGGTAAGCTAGAGAAGTTAAAGGACGCTTTCGATATTGCTGTAGAAGGAGTGCTTAAAAGAGTGGAACGTGTTCGAAGAGGACTTGAGGGTGACCAAGGTTTTGGTGTGGAGCAGACGTGGAAACAGGTGTTACCCGAAGGGTCCTACGAAGAGGATTGGGACAGATTTTTTAAGCAGTTCGAGTTCCGAAGTTCGTGAGACCACGAAGTG
>AtMBGT1_synthesised_gene
CACTCTGTGGTCTCAAATGCGACCCAAGAATTATTCTCAGATGGAGAAACCCATCTCCATCACGACGGGGAAGTTTCGGACCAACAACAACAACAATCATAACAATGTCTGGTTCGTTGTTCCACTCTTCTTTATCCTCTGTTTCGTTCTCCTCTGTTTCGACTACTCTGCTCTCTTCACCGACACAGACGAAACCGCTTTCTCAATTCCCGACGTTACCCAGAAATCAACATCCTCTGAATTCACGAAAGATGACAACTTTTCTCGATTTCCCGACGACCCATCTCCTGATTCTTCCTGCTCCGGTCGGTATATTTACGTTCATGAACTTCCGTATAGATTCAATGGTGATTTGCTTGATAATTGCTTTAAGATTACTAGAGGAACCGAGAAAGATATTTGTCCTTACATCGAAAACTATGGTTTTGGTCCTGTCATTAAGAATTACGAGAATGTGTTGTTGAAACAAAGTTGGTTTACGACCAATCAGTTCATGCTCGAGGTGATATTCCACAACAAGATGATAAATTACAGATGTTTGACTAATGATTCGTCACTAGCGTCTGCGGTTTTCGTGCCGTTTTACGCAGGTCTTGATATGAGTAGGTATCTTTGGGGTTTCAACATTACGGTTAGAGATTCTTCGTCCCATGAGCTGATGGATTGGCTTGTGGTACAGAAAGAGTGGGGTCGAATGTCTGGTAGAGATCATTTCTTGGTTTCAGGAAGGATTGCTTGGGATTTCAGGAGACAGACTGATAACGAATCTGATTGGGGAAGCAAGCTTCGGTTCTTACCCGAGTCGCGGAACATGTCGATGTTGTCCATCGAATCGAGTTCTTGGAAGAATGATTACGCTATTCCGTATCCGACTTGTTTCCATCCAAGATCAGTGGATGAGATTGTGGAGTGGCAGGAGCTAATGAGGTCCCGAAAACGCGAGTATCTATTCACCTTTGCGGGTGCTCCAAGGCCTGAATATAAAGACTCGGTTCGAGGGAAGATTATAGATGAGTGTTTGGAGTCAAAGAAGCAATGTTACTTGCTAGACTGTAACTATGGGAATGTGAATTGTGACAACCCTGTCAATGTGATGAAGGTTTTCAGGAACTCGGTGTTTTGTCTCCAGCCCCCGGGTGATTCTTACACTAGAAGGTCAATGTTTGATTCAATATTGGCAGGCTGCATTCCGGTGTTCTTCCATCCAGGGACAGCGTACGCTCAATACAAGTGGCATTTGCCCAAGAATCACTCGAGTTACTCGGTTTATCTACCGGTTAAGGATGTGAAGGAGTGGAATATCAAAATCAAGGAGAGGTTGATTGAGATTCCAGAGGAAAGAGTGGTGAGGTTGAGAGAAGAGGTTATAAGGTTGATACCGAAGGTTGTCTACGCTGATCCGAAGTACGGATCGGATGGGAGTGAAGATGCATTTGAGTTGGCTGTGAAGGGAATGTTGGAGAGGATTGAAGAAGTGAGAGAGATGATGAGACAAGGGAAAGATGGTAGTGATGGGTTTGATGATAGAGATGATTATAAGTATACTTTTTCTCCATATGAAGAACCTCAAGTTTTGGCTAGTTCGTGAGACCACGAAGTG

## Slide 6
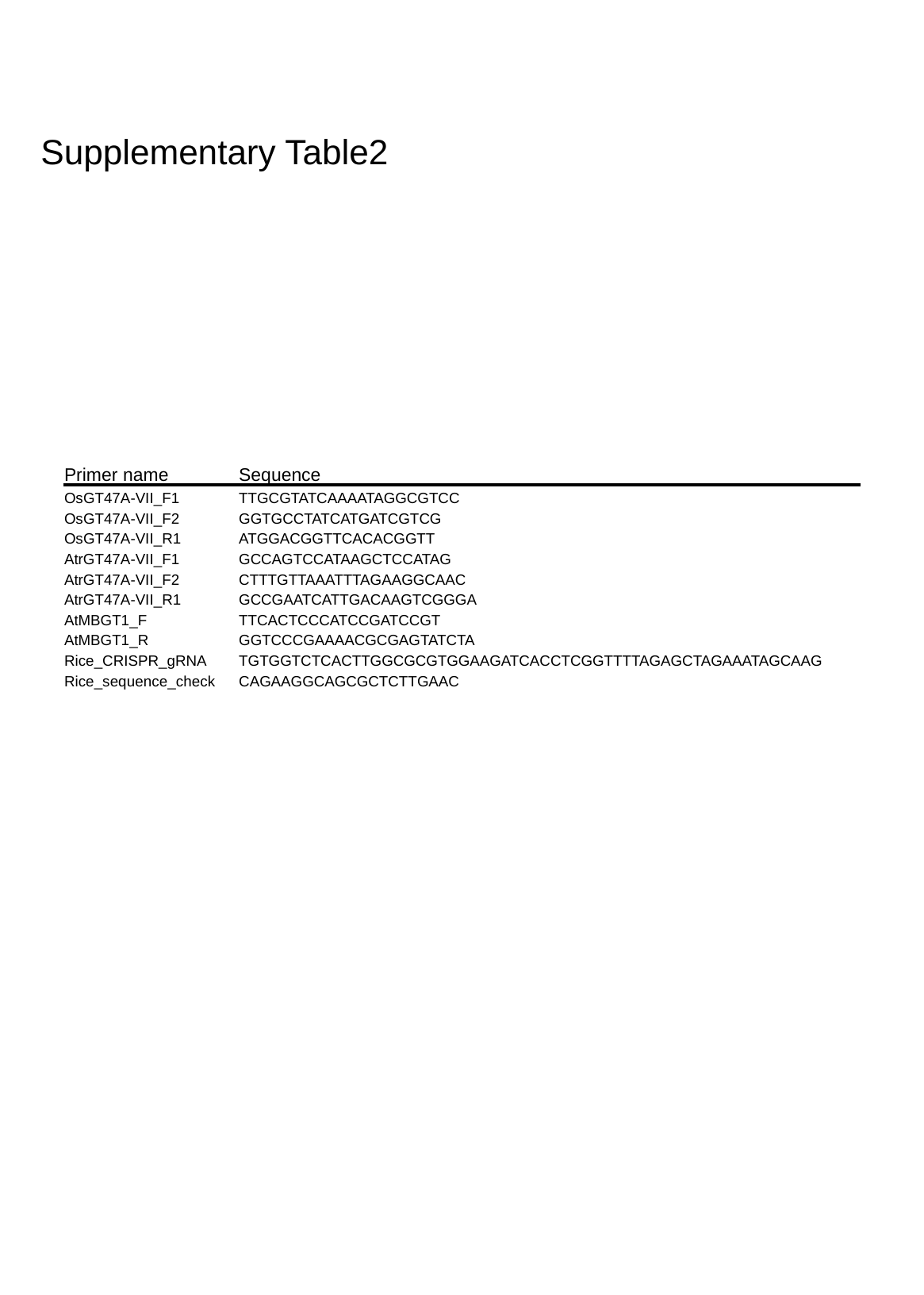

Supplementary Table2
| Primer name | Sequence |
| --- | --- |
| OsGT47A-VII\_F1 | TTGCGTATCAAAATAGGCGTCC |
| OsGT47A-VII\_F2 | GGTGCCTATCATGATCGTCG |
| OsGT47A-VII\_R1 | ATGGACGGTTCACACGGTT |
| AtrGT47A-VII\_F1 | GCCAGTCCATAAGCTCCATAG |
| AtrGT47A-VII\_F2 | CTTTGTTAAATTTAGAAGGCAAC |
| AtrGT47A-VII\_R1 | GCCGAATCATTGACAAGTCGGGA |
| AtMBGT1\_F | TTCACTCCCATCCGATCCGT |
| AtMBGT1\_R | GGTCCCGAAAACGCGAGTATCTA |
| Rice\_CRISPR\_gRNA | TGTGGTCTCACTTGGCGCGTGGAAGATCACCTCGGTTTTAGAGCTAGAAATAGCAAG |
| Rice\_sequence\_check | CAGAAGGCAGCGCTCTTGAAC |

## Slide 7
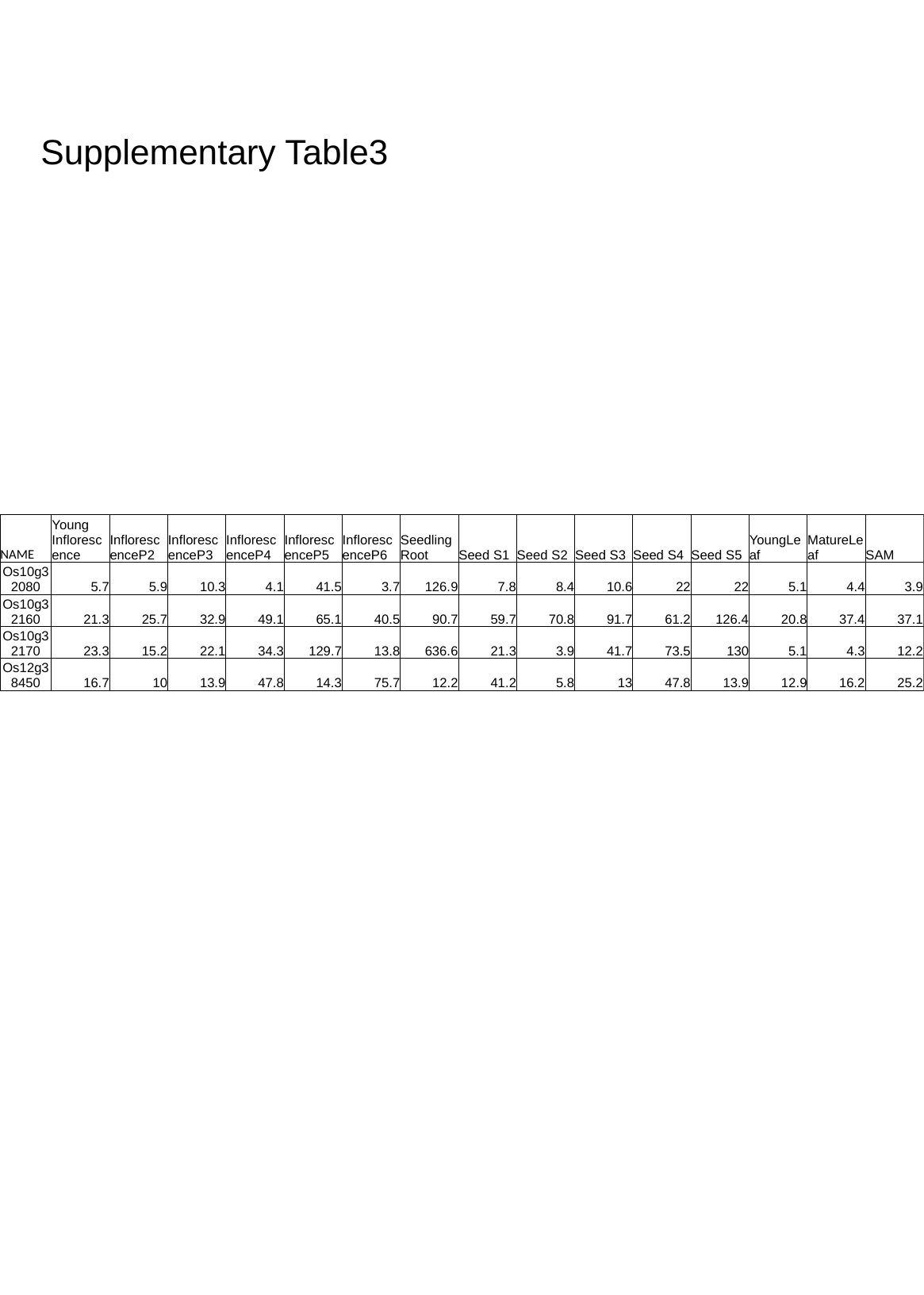

Supplementary Table3
| NAME | Young Inflorescence | InflorescenceP2 | InflorescenceP3 | InflorescenceP4 | InflorescenceP5 | InflorescenceP6 | Seedling Root | Seed S1 | Seed S2 | Seed S3 | Seed S4 | Seed S5 | YoungLeaf | MatureLeaf | SAM |
| --- | --- | --- | --- | --- | --- | --- | --- | --- | --- | --- | --- | --- | --- | --- | --- |
| Os10g32080 | 5.7 | 5.9 | 10.3 | 4.1 | 41.5 | 3.7 | 126.9 | 7.8 | 8.4 | 10.6 | 22 | 22 | 5.1 | 4.4 | 3.9 |
| Os10g32160 | 21.3 | 25.7 | 32.9 | 49.1 | 65.1 | 40.5 | 90.7 | 59.7 | 70.8 | 91.7 | 61.2 | 126.4 | 20.8 | 37.4 | 37.1 |
| Os10g32170 | 23.3 | 15.2 | 22.1 | 34.3 | 129.7 | 13.8 | 636.6 | 21.3 | 3.9 | 41.7 | 73.5 | 130 | 5.1 | 4.3 | 12.2 |
| Os12g38450 | 16.7 | 10 | 13.9 | 47.8 | 14.3 | 75.7 | 12.2 | 41.2 | 5.8 | 13 | 47.8 | 13.9 | 12.9 | 16.2 | 25.2 |

## Slide 8
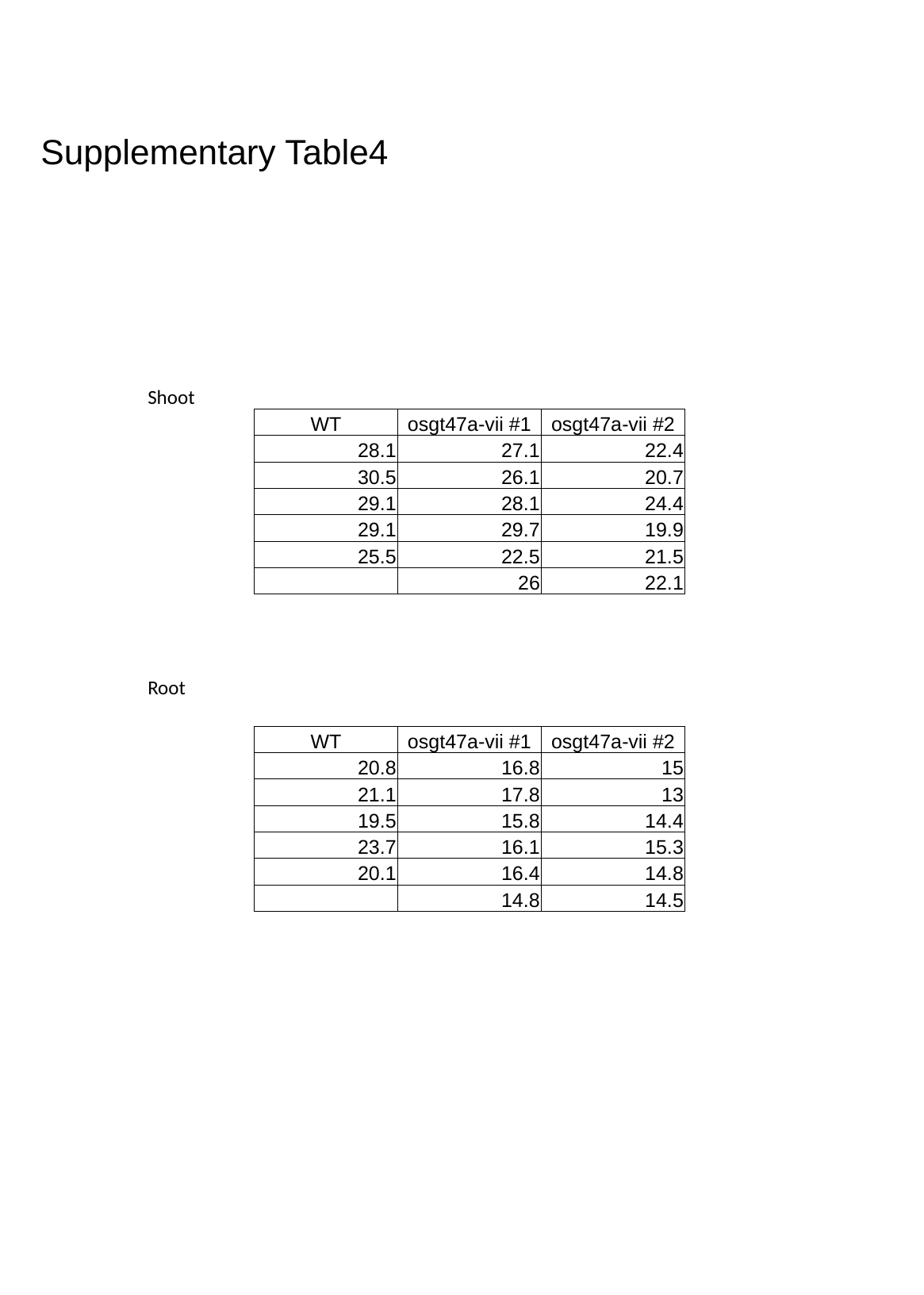

Supplementary Table4
| Shoot | | | |
| --- | --- | --- | --- |
| | WT | osgt47a-vii #1 | osgt47a-vii #2 |
| | 28.1 | 27.1 | 22.4 |
| | 30.5 | 26.1 | 20.7 |
| | 29.1 | 28.1 | 24.4 |
| | 29.1 | 29.7 | 19.9 |
| | 25.5 | 22.5 | 21.5 |
| | | 26 | 22.1 |
| | | | |
| | | | |
| | | | |
| Root | | | |
| | | | |
| | WT | osgt47a-vii #1 | osgt47a-vii #2 |
| | 20.8 | 16.8 | 15 |
| | 21.1 | 17.8 | 13 |
| | 19.5 | 15.8 | 14.4 |
| | 23.7 | 16.1 | 15.3 |
| | 20.1 | 16.4 | 14.8 |
| | | 14.8 | 14.5 |
